# The Impact of Acute Care Surgery Model on the Management of Acute Appendicitis and Cholecystitis: A Single-Center Study

**DOI:** 10.7759/cureus.26724

**Published:** 2022-07-10

**Authors:** Ibrahim Al Babtain, Sondus A Alraee, Mishary M Shalhoub, Leen O Hijazi, Arwa A albalawi, Modhi alamer

**Affiliations:** 1 Department of General Surgery, King Abdulaziz Medical City Riyadh, Riyadh, SAU; 2 Medicine and Surgery, King Saud bin Abdulaziz University for Health Sciences College of Medicine, Riyadh, SAU; 3 General Surgery, King Saud University, Riyadh, SAU

**Keywords:** surgery, emergency, cholecystectomy, appendectomy, acute care surgery

## Abstract

Background

Acute care surgery (ACS) is a novel model for the provision of emergency general surgery (GS) care. Investigating the impact of the ACS team on the management of acute emergencies can help in establishing proper management measures and improving patient care in an emergency setting. The study aims to compare the performance indicators and patient outcomes such as hospital length of stay (LOS), time to diagnosis, and operation before and after the implementation of the acute care system.

Methods

The study reviewed two retrospective cohorts: the pre-ACS system (n = 202) from January 2012 to December 2013 and the post-ACS system (n = 188) from January 2014 to December 2015, which were done in a tertiary care center. All adult patients diagnosed with acute appendicitis and cholecystitis requiring emergency surgery were included.

Results

There was an improvement in the time interval between GS referral to the diagnosis of acute appendicitis and cholecystitis (p = 0.07) and from diagnosis to the start of the operation (p = 0.38). Patients in the post-ACS model had a shorter hospital stay than the pre-ACS model patients with [M = 3.69 SD(3.18) days versus M = 3.57 SD (3.60) days, p = 0.25]. Time from the emergency department arrival to GS referral did not show an improvement [M = 4.36 SD(3.34)] hours in the pre-ACS model versus [M = 4.53 SD(3.98)] hours in the ACS model, p = 0.86).

Conclusion

The ACS model led to earlier diagnosis of acute appendectomy and cholecystectomy cases and reduced the LOS. The introduction of the ACS model in Saudi Arabia showed improvement in patient care during acute emergencies. Further studies including multiple centers with larger sample sizes and longer review periods are needed to evaluate the efficiency and cost-effectiveness of the ACS model.

## Introduction

The acute care surgery (ACS) model of care is a novel paradigm in the provision of emergency general surgery (GS) [1]. Traditionally, non-traumatic patients presenting to the emergency department with acute surgical conditions were seen by the general surgeon-on-call [2]. Patients requiring surgery were then added to the general emergency list and had to compete for an operating room with patients who were already booked from all other specialties [2]. As the surgeon-on-call is occupied with other commitments such as elective surgeries and outpatient clinics booked for the same day, patients who required surgery often had to wait hours for their operation to start [2]. Reported disadvantages of the traditional model include cancellation or rescheduling of elective surgeries due to the increase in the acute surgical caseload, unavailability of operating rooms, and delay of urgent surgeries, which negatively affect the patient outcome and increase the burden on the responsible surgeon [3].

In our institution, both models of care are led by consultants and consist of a consultant general surgeon, an assistant consultant, a senior, and a junior resident. All the team members are involved in the early decision-making. In the pre-ACS model, the surgical team covers the acute surgical presentations, elective surgery caseload, outpatient clinics, and rounds in a 24-hour on-call shift. The 24-hour on-call shift consists of in-house morning hours for the consultant and the residents and home on-call during the night hours for the consultants only. The way the ACS model is implemented in our institution differs from the way it is implemented in Canadian and American hospitals. In our institute, the ACS team covers the acute care surgical cases from 8 am to 5 pm from Sunday to Wednesday and 24 hours on Thursday. An on-call shift covers the acute care cases from 5 pm to 8 am each day with a different team. During the weekends, another surgical team is on call. The ACS model was implemented in January 2014 at a tertiary care facility in Riyadh, Saudi Arabia. The potential benefit of the ACS model is that acute surgical emergencies can be predicted and planned for ahead of time, and dedicated operating rooms and surgical teams can be provided [3]. This would prevent any delay in patient care and would allow less interference with the elective surgery schedule [2].

We aim to investigate the impact of the ACS team on the management of patients presenting with acute appendicitis and acute cholecystitis, two of the most common causes of acute surgical presentations. We compared the performance indicators such as the time intervals for treatment and patient outcomes prior to and after the introduction of the ACS team.

## Materials and methods

A retrospective study of medical records was conducted in a level 1 trauma center in Riyadh, Saudi Arabia, following a local Institutional Review Board (IRB) approval. The study was conducted in the Department of General Surgery, and data over four years were used in the study. It included two years before the introduction of the ACS system from January 2012 to December 2013 and two years after the introduction of the ACS system from January 2014 to December 2015. Patients of both genders aged 14 years and older who are considered the adult population in our institute and diagnosed with acute cholecystitis and appendicitis presenting at the emergency department were included in the study. Patients who underwent cholecystectomy and appendectomy as a scheduled procedure or as part of another major operation were excluded from the study. Also, patients with associated complications like in case of appendicular mass, choledocholithiasis, cholangitis, or pancreatitis who were treated conservatively were excluded from the study. These complicated conditions require other procedures that could independently modify the hospital length of stay (LOS) and treatment outcomes and confound the analysis. A total of 390 patients who met the inclusion criteria were included in the study.

Information obtained from the medical records included the patient's age, gender, body mass index (BMI), chronic diseases like hypertension and diabetes, the time intervals from emergency department (ED) arrival to surgical consult request, time from the consultation to diagnosis, time from diagnosis to operation, LOS, duration of the operation, type of the procedure: open or laparoscopic, intraoperative conversion to open surgery, and postoperative complications. The primary outcome of the study was to compare the key time intervals between pre-ACS and ACS models.

All variables were entered and analyzed in Statistical Analysis System (SAS), version 9.0 (SAS Institute, Cary, North Carolina). The data were presented in frequencies and percentages or means with standard deviations. Wilcoxon two-sample test was used to compare the numerical data. Nominal data were compared using the chi-square test and Fisher's exact test as needed.

## Results

Data from 390 patients were extracted and categorized into two groups: the pre-ACS (n = 202) group and the ACS group (n = 188). There was no significant difference in demographic characteristics, medical history of the patients, and emergency surgeries between the two groups as mentioned in Table 1. The mean age of the pre-ACS and ACS model was [M = 29.46 SD(13.80)] versus [M = 31.32 SD(15.03)], respectively. The number of emergency appendectomies was higher than the number of emergency cholecystectomies in both groups.

**Table 1:** Comparison of patient demographics, medical history, and emergency surgery performed between pre-ACS model and ACS model ACS: Acute care surgery.

Demographics	Pre-ACS model (n = 202)	ACS model (n = 188)
Age mean (SD)	29.46 (13.80)	31.32 (15.03)
Sex, n (%)		
Male	114 (29.23)	112 (28.72)
Female	88 (22.56)	76 (19.49)
BMI mean (SD)	26.33 (6.16)	26.73 (5.98)
Diabetes, n (%)	17 (4.36)	20 (5.13)
Hypertension, n (%)	16 (4.10)	15 (3.85)
Abdominal surgery, n (%)		
Appendectomies, n (%)	164 (42.05%)	156 (40.00%)
Cholecystectomies, n (%)	38 (9.74%)	32 (8.21%)
Previous abdominal surgery, n (%)	27 (6.94%)	28 (7.20%)

A comparison of the overall performance indicators for the pre-ACS and ACS models is shown in Table 2. Time from the ED arrival to GS referral did not show an improvement (4.36 hours in the pre-ACS model versus 4.53 hours in the ACS model). Although there was a trend of some improvement in the time interval between GS referral to GS diagnosis and GS diagnosis to the start of the operation, the differences were not significant. ACS model patients had a shorter hospital LOS than pre-ACS model patients [M = 3.69 SD(3.18) days versus M = 3.57 SD(3.60) days, p = 0.25]. The postoperative LOS in the pre-ACS and ACS models did not show any significant difference [M = 2.22 SD(2.24) versus M = 2.51 SD(2.85), p = 0.61].

**Table 2:** Comparison of important time intervals between the pre-ACS and ACS models ACS: Acute care surgery; ED: Emergency department; GS: General surgery; LOS: Length of stay.

Time intervals mean (SD)	Pre-ACS model (n = 202)	ACS model (n = 188)	P-values
Time from ED arrival to GS referral (h)	4.36 (3.34)	4.53 (3.98)	0.86
Time from GS referral to diagnosis (h)	9.99 (12.63)	7.92 (10.75)	0.07
Time from diagnosis to operation start (h)	25.41 (48.84)	19.12 (34.76)	0.38
Total admission LOS (days)	3.69 (3.18)	3.57 (3.60)	0.25
Postoperative LOS (days)	2.22 (2.24)	2.51 (2.85)	0.61

A summary of the operative details of pre- and post-ACS models is shown in Table 3. Under the pre-ACS model, the mean (SD) of operation duration was 87.28 (43.64) and 94.78 (53.50) in the ACS model. The most common procedure for both groups was a laparoscopic appendectomy. The number of identified complications after surgery was four (1.03%) in the pre-ACS model and one (0.26%) in the ACS model.

**Table 3:** Comparison of operative details and complications between the pre-ACS and ACS models ACS: Acute care surgery.

Details of the operation performed	Pre-ACS (n = 202)	ACS model (n = 188)
Duration of operation (min)	87.28 (43.64)	94.78 (53.50)
Operation type, n (%)		
Laparoscopic	183 (46.92%)	180 (46.15%)
Open	18 (4.62%)	7 (1.79%)
Conversion of laparoscopic to open, n (%)	1 (0.26%)	1 (0.26%)
Complications, n (%)	4 (1.03%)	1 (0.26%)

## Discussion

Acute appendicitis and acute cholecystitis are considered common causes of the acute abdomen [4]. Globally, the incidence of appendectomy during the 21st century was 100 per 100,000 person-years [5]. Therefore, appendectomies and cholecystectomies are considered emergency surgeries that are frequently encountered by surgeons [4]. In our study, we chose these two conditions to compare the performance indicators and outcomes of the pre-ACS and ACS models. In 2014, our hospital introduced the ACS model with the purpose of providing patient care in a timely manner. Any improvement in patient care would be identified by a reduction in time to diagnosis, time to operation, rate of complication, and the hospital LOS.

The introduction of an ACS system has resulted in improved efficiency and patient outcomes for operative management of acute appendicitis and cholecystitis. The time required from the ED arrival to surgical referral was not significantly changed after the introduction of the ACS model [M = 4.36 SD(3.34) versus M = 4.53 SD(3.9) hours, p = 0.86] because the patients were assessed by the ED consultants initially and then they were referred to the ACS team. The long time period in both models between the diagnosis and operation is probably due to the large number of patients coming to the level 1 trauma center and the crowding in the ED. However, the time between GS consultation request to definitive diagnosis [M = 9.99 SD(12.63) versus M = 7.92 SD(10.75), p = 0.07] and the time from diagnosis to operation start [M = 25.41 SD(48.84) versus M = 19.12 SD(34.76), p = 0.38] were improved after the implantation of the ACS model. Similar results have been reported by Fu et al. and Qureshi et al. where both studies showed an improvement in time from ED consultation request to operation start [6,7]. Surgeons were accommodated into a well-structured and dedicated service, eliminating other clinical duties that allowed the surgeons to be immediately available. However, the after-hour on-call team did not perform all of the surgeries immediately at presentation; some surgeries were delayed until the next morning. One possible reason for this delay is that in both models, the salaries are fixed; the number of surgeries performed did not affect the monthly income. The lack of motivation to perform surgeries at night on-call hours might have led to some delaying of cases to the next morning. Postponing the cases overwhelmed the surgeons-on-call on the next day. This might explain the minimal improvement shown in our results.

Previous studies have shown mixed results in regard to the total hospital LOS and the postoperative LOS of patients with acute appendicitis and cholecystitis in the ACS model [6,8,9]. Some studies only measured the total hospital LOS and some only mentioned the postoperative LOS. In our study, we measured the total LOS and the postoperative LOS, both of which did not show any statistical significance or major change in the number of days. This could be due to some factors that could have an influence on LOS. These factors include the pre-surgery admission time that depends on the patient flow in the wards, availability of beds, and operation theaters. This can also explain the mixed results seen in the literature. The presence of comorbidities that need to be controlled before the surgery like diabetes and hypertension may also have an effect on the LOS in both the pre-ACS and ACS models.

Almost all appendectomies in both models of care were performed by the laparoscopic approach. The presence of more complications in the pre-ACS model could be attributed to the fact that the surgeons on a 24-hour on-call shift are more likely to be burned out, which might affect the quality of care. Also, consultant surgeons were not always present at the hospital to guide the residents during surgery as they were on home on-call. Our study has several limitations. The current study was done in a single center, including a small number of patients and a short period of review after the implementation of the ACS model.

## Conclusions

We investigated the impact of the ACS model on the outcomes of management of acute appendicitis and cholecystitis. The introduction of the ACS model showed improvement in the performance indicators and patient outcomes. The limitations we came across in this study were the small sample size and it being a single-center study. Further studies including multiple centers with larger sample sizes and longer review periods are needed to evaluate the efficiency and cost-effectiveness of the ACS model. Also, studies should assess the surgeon’s satisfaction with the ACS model implementation.
